# Ultrathin bismuth nanosheets from in situ topotactic transformation for selective electrocatalytic CO_2_ reduction to formate

**DOI:** 10.1038/s41467-018-03712-z

**Published:** 2018-04-03

**Authors:** Na Han, Yu Wang, Hui Yang, Jun Deng, Jinghua Wu, Yafei Li, Yanguang Li

**Affiliations:** 1 Institute of Functional Nano and Soft Materials (FUNSOM), Jiangsu Key Laboratory for Carbon-Based Functional Materials and Devices, Soochow University, Suzhou, 215123 China; 2 College of Chemistry and Materials Science, Nanjing Normal University, Nanjing, 210023 China

## Abstract

Electrocatalytic carbon dioxide reduction to formate is desirable but challenging. Current attention is mostly focused on tin-based materials, which, unfortunately, often suffer from limited Faradaic efficiency. The potential of bismuth in carbon dioxide reduction has been suggested but remained understudied. Here, we report that ultrathin bismuth nanosheets are prepared from the in situ topotactic transformation of bismuth oxyiodide nanosheets. They process single crystallinity and enlarged surface areas. Such an advantageous nanostructure affords the material with excellent electrocatalytic performance for carbon dioxide reduction to formate. High selectivity (~100%) and large current density are measured over a broad potential, as well as excellent durability for >10 h. Its selectivity for formate is also understood by density functional theory calculations. In addition, bismuth nanosheets were coupled with an iridium-based oxygen evolution electrocatalyst to achieve efficient full-cell electrolysis. When powered by two AA-size alkaline batteries, the full cell exhibits impressive Faradaic efficiency and electricity-to-formate conversion efficiency.

## Abstract (web summary)

The electroreduction of carbon dioxide to liquid products provides an appealing method to convert atmospheric carbon into valuable fuels. Here, the authors perform a topotactic transformation of bismuth oxyiodide to bismuth nanosheets that act as highly selective CO_2_-to-formate electrocatalysts.

## Introduction

Electrocatalytic CO_2_ reduction to useful chemical fuels represents an attractive route to the capture and utilization of atmospheric CO_2_^1,2^. When coupled with renewable energy sources such as solar energy, this process could potentially enable a sustainable energy economy and chemical industry^3,4^. One key technological challenge in this process remains the development of active, durable and selective electrocatalysts for CO_2_ reduction reaction (CO_2_RR)^5–12^. Formate (or formic acid) is a common CO_2_RR product. It is an important chemical intermediate in many industrial processes, and can be used as the chemical fuel in direct formate (or formic acid) fuel cells^13,14^. In 1980s, Hori et al. first reported that several II B, III A, and IV A metals (Pb, Cd, Hg, In, Sn, and Tl) could reduce CO_2_ to formate^15–17^. They have high hydrogen overpotentials, and weak affinity toward CO_2_^•−^ intermediate, which then tends to be protonated at the carbon atom and ultimately transforms to formate as the major reduction product^17^. Unfortunately, many of these heavy metals (Pb, Cd, Hg and Tl) are highly toxic and environmentally hazardous, and therefore are out of consideration for practical applications. Sn-based materials have gained major interest for formate production, but usually suffer from limited reaction selectivity (peak selectivity 50 ~ 80%) accompanied by the significant cogeneration of H_2_ and CO^18–20^.

Bi locates close to traditional formate-producing metals in the periodic table. It is therefore suggested to be also active for CO_2_ reduction to formate, yet is significantly less toxic and more environmentally benign than many of its neighbors. Previous studies on Bi-based materials were mostly conducted in ionic liquids or aprotic electrolytes with CO as the end product^21–23^. Its potential for CO_2_RR in aqueous solution just starts to be unveiled^24–26^. To further improve its performance would require structural engineering at the nanoscale to enlarge its surface areas. Bi is consisted of stacked layers of buckled honeycomb structure similar to black phosphorus. This permits Bi to be potentially exfoliated to its two-dimensional (2D) mono- or few-layers with enlarged surface area and enhanced electrochemical activity. Stable Bi monolayer (i.e., bismuthene) was in fact theoretically predicted^27^. Nevertheless, the direct preparation of Bi mono- or few-layers via conventional top-down (mechanical exfoliation) or bottom-up (chemical synthesis) methods is highly challenging, and successful examples are very sparse in literature as far as we know.

We report here an indirect approach to prepare ultrathin Bi nanosheets via in situ topotactic transformation of bismuth oxyiodide (BiOI) naonsheet template. Most remarkably, the 2D morphology is largely preserved during the structural transformation of Bi atoms. These ultrathin Bi nanosheets can act as an efficient CO_2_RR electrocatalyst for the conversion of CO_2_ to formate with impressive activity and selectivity close to 100% over a broad potential window, as well as satisfactory durability.

## Results

### Topotactic reduction from BiOI nanosheets to Bi nanosheets

**Fig. 1:**
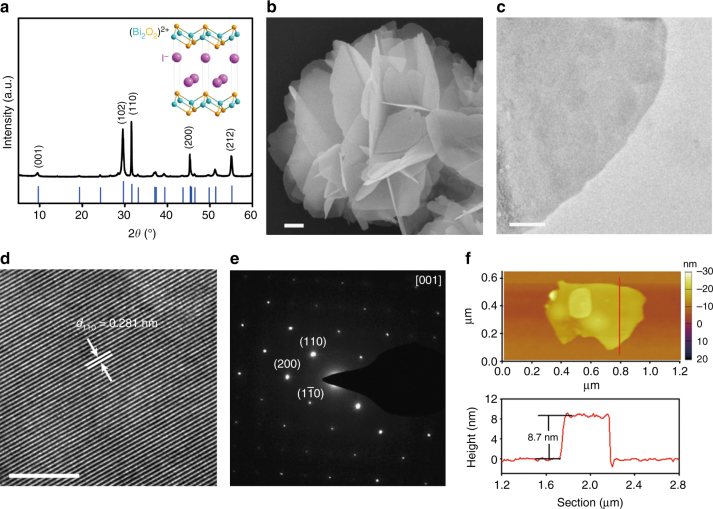
Structural characterizations of BiOI nanosheets. **a** XRD pattern and (inserted) schematic crystal structure; **b** SEM image; **c**, **d** TEM images at different magnifications; **e** SAED pattern; **f** AFM image and corresponding height profile. Scale bar, 200 nm (**b**); 50 nm (**c**); 5 nm (**d**)

We started with the preparation of BiOI nanosheets. As shown in the insert of Fig. 1a, BiOI possesses a layered tetragonal structure where [Bi_2_O_2_] slabs are sandwiched between anionic iodide layers. A facile hydrothermal method was adopted here for its synthesis by reacting Bi(NO_3_)_3_ and KI at 160 °C for 2 h (see Methods for details). Figure 1a shows the X-ray diffraction (XRD) pattern of as-prepared product. All the diffraction peaks were assignable to tetragonal BiOI. Based on the (001) peak width, we estimated that the thickness along the *c*-direction was ~16 nm. Scanning electron microscopy (SEM) image of the as-prepared product showed that it was comprised of hierarchical microflowers that were assembled from very thin nanosheets oriented to different angles (Fig. 1b). The nanosheet thickness was so small that they were almost transparent to the electron beam. To better understand the microstructure, we broke BiOI microflowers into individual nanosheets by gentle sonication, and then examined them under transmission electron microscopy (TEM). As shown in Fig. 1c, these nanosheets had uniform contrast. High-resolution TEM study revealed obvious lattice fringes corresponding to the (110) plane of tetragonal BiOI (Fig. 1d). Selected area electron diffraction (SAED) pattern over a large piece of nanosheet displayed a single set of diffraction spots with a fourfold symmetry (Fig. 1e). Its zone axis was found to be along the *c*-direction, indicating that BiOI nanosheets were terminated with the relatively stable (001) plane. Furthermore, BiOI nanosheet pieces were analyzed by atomic force microscopy (AFM) (Fig. 1f). From a typical height profile as shown, the nanosheet thickness was measured to be ~8.7 nm—corresponding to 9–10 layers. The influences of different synthetic parameters on the product morphology were also explored and summarized in Supplementary Fig. 1.

**Fig. 2:**
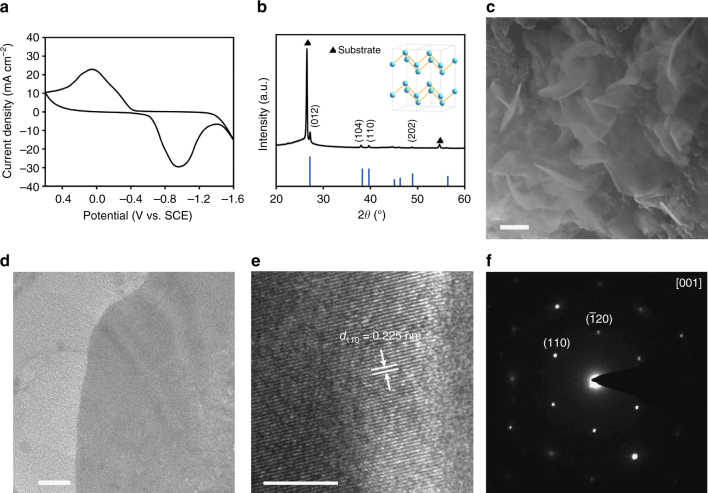
Structural characterizations of topotactically reduced BiNS. **a** CV curve of BiOI showing its reduction to metallic Bi at cathodic potentials; **b** XRD and (inserted) schematic crystal structure of Bi; **c** SEM image; **d**, **e** TEM images at different magnifications; **f** SAED pattern of BiNS. Scale bar, 200 nm (**c**); 20 nm (**d**); 5 nm (**e**)

BiOI is stable under ambient conditions, but undergoes a reductive transformation at cathodic potentials. We attempted to electrochemically reduce BiOI and interrogate the composition and microstructure of its reduced counterpart. As-prepared BiOI was loaded onto carbon fiber paper as the working electrode. Figure 2a shows the typical cyclic voltammetry (CV) curve of BiOI in 0.5 M NaHCO_3_ electrolyte. It exhibited a pair of pronounced and roughly symmetric redox waves between −1.4 ~ 0.6 V versus saturated calomel electrode (SCE) as contributed by the reversible interconversion between Bi^3+^ and metallic Bi (supported by the Pourbaix diagram of Bi in H_2_O^28^). Reduced material supported on the working electrode was taken out of the electrolyte, briefly rinsed with distilled water, and immediately subjected to spectroscopic and microscopic characterizations. Its XRD measurement unveiled diffraction peaks assignable to rhombohedral Bi, overlaid on the top of intense signals from the carbon fiber paper substrate (Fig. 2b). There was no detectable signal from original tetragonal BiOI phase suggesting the complete conversion from BiOI to metallic Bi. X-ray photoelectron spectroscopy (XPS) analysis of the reduced product indicated that its surface was free of iodine (Supplementary Fig. 2). Most remarkably, this reductive transformation did not seem to significantly compromise or degrade the microstructure despite the considerably different atomic arrangements between tetragonal BiOI and rhombohedral Bi. SEM image showed that the reduced Bi retained the 2D nanosheet morphology (accordingly denoted as BiNS hereafter, Fig. 2c). Compared to the BiOI nanosheet template, BiNS appeared to be even thinner and more transparent to electron beam. Some nanosheets were curved or folded, presumably due to the soft nature of metallic Bi. TEM image of BiNS showed that reduced nanosheets had no sign of pulverization (Fig. 2d). High-resolution TEM analysis revealed obvious lattice fringes with 60° intersection angle that corresponded to (110) planes of rhombohedral Bi (Fig. 2e). SAED pattern over an entire sheet showed a single set of diffraction spots with a sixfold symmetry. Its zone axis was indexed along [001] direction (Fig. 2f).

Above microscopic results corroborated that BiNS preserved its 2D structure integrity and single crystallinity, and the reductive transformation from layered tetragonal BiOI to layered rhombohedral Bi was likely topotactic with [001]_BiOI_//[001]_Bi_. To better understand this topotactic transformation, we further compare their crystal structures (Supplementary Fig. 3a). Despite the different arrangements of Bi atoms within each layer, they actually shared similar lattice parameters: *a’* = *b’* = 8.06 Å for 2 × 2 supercell (8 atoms) of BiOI and *a’* = 7.97 Å and *b’* = 9.20 Å for √2 × 2√2 supercell (8 atoms) of Bi. The conversion from tetragonal BiOI to rhombohedral Bi could be achieved by simply sliding some Bi atoms along the *b’*-direction as shown in Supplementary Fig. 3b, which involved no significant volume change and no appreciable energy barrier according to our density functional theory (DFT) simulation (Supplementary Fig. 3c). Furthermore, we performed first-principles molecular dynamic (FPMD) simulations using an optimized 6 × 6 supercell of tetragonal Bi at 300 K, and found that a large number of hexatomic Bi rings form after 1000 steps (1 ps) (Supplementary Fig. 3d). It suggested that once reduced, the conversion from BiOI to rhombohedral Bi was energetically favored and kinetically fast. Ultrathin nanosheets of metallic Bi presented an ideal material for CO_2_RR electrocatalysis due to its enlarged surface area. We estimated the surface active Bi sites by integrating the cathodic peak area from the CV curve, and found that it accounted for ~12% of total Bi sites (Supplementary Fig. 4).

### CO_2_RR performance of BiNS

**Fig. 3:**
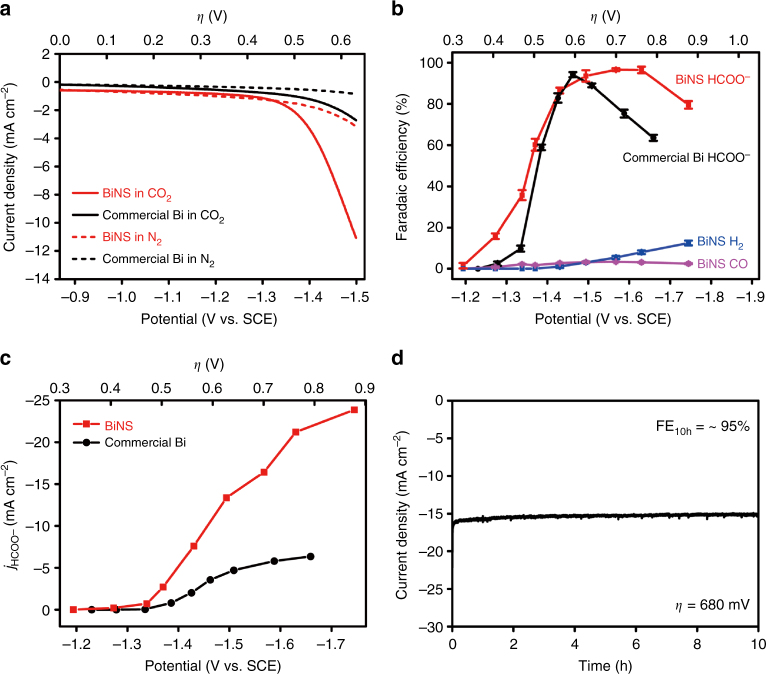
Electrochemical measurements of BiNS. **a** Polarization curves of BiNS and commercial Bi nanopowder in N_2_- or CO_2_-saturated 0.5 M NaHCO_3_; **b** potential-dependent Faradaic efficiencies of HCOO^−^, CO, and H_2_ on BiNS in comparison with the Faradaic efficiency of HCOO^−^ on commercial Bi nanopowder; **c** potential-dependent HCOO^−^ partial current density on BiNS and commercial Bi nanopowder; **d** amperometric (*i* ~ *t*) stability of BiNS at *η* = 680 mV for 10 h

We next investigated the CO_2_RR performance of topotactically reduced BiNS within a gas-tight two-compartment electrochemical cell (see Methods for details). Its polarization curve in CO_2_-saturated 0.5 M NaHCO_3_ exhibited a cathodic current onset at approximately −1.3 V versus SCE due to CO_2_RR, and beyond that, the current density continuously increased and reached 11 mA cm^−2^ at −1.5 V (Fig. 3a). Such an activity was markedly enhanced over commercial Bi nanopowder measured under identical conditions. Control experiments suggested that in the absence of any CO_2_ feed gas, hydrogen evolution reaction (HER) became the dominant cathodic process, and its current density was much diminished—in line with the poor HER activity of Bi. In order to identify and quantify the reduction products, electrolysis was performed at different potentials between −1.2 and −1.8 V for 2 h. Resultant gaseous products were periodically sampled and examined using gas chromatography (GC), and the liquid products were analyzed by nuclear magnetic resonance (NMR) spectroscopy and ion chromatography (IC) at the end of each electrolysis. We found that formate was the dominant reduction product, accompanied by the cogeneration of a small amount of CO and H_2_. Faradaic efficiency of different products at various potentials were calculated (see Methods for details) and summarized in Fig. 3b. Formate was first reliably and reproducibly detected at as positive as −1.27 V, translating to a small overpotential of 0.4 V. Initially, its Faradaic efficiency was ~16%, then quickly rose to >95% at ~−1.5 V, and maintained close to 100% until −1.7 V. Faradaic efficiency for the two gaseous products remained small (<5%) at low-to-medium overpotentials. Only beyond −1.7 V was a considerable amount of H_2_ detected presumably because CO_2_RR became diffusion-limited under this condition. By contrast, the Faradaic efficiency for formate on commercial Bi nanopowder took off at −1.33 V, reached the peak value at −1.46 V and then started to decline at potentials negative to −1.5 V. Furthermore, the formate partial current density of BiNS and commercial Bi nanopowder was calculated and plotted against the working potential as shown in Fig. 3c. The former delivered a maximum value of *j*_HCOO−_ = 24 mA cm^−2^ at −1.74 V, whereas that of the latter did not exceed 6 mA cm^−2^. Their mass-specific current density was also compared in Supplementary Fig. 5. The combination of large catalytic current density and high-formate selectivity over a broad potential observed for BiNS was highly desirable for practical applications, and attributed to its advantageous nanostructure with enlarged surface area and abundant under-coordinated Bi sites. It placed our electrocatalyst on the top of Bi-based CO_2_RR electrocatalysts^24,25,29^. Noteworthy was that BiNS also outperformed most Sn-based CO_2_RR electrocatalysts, which were more frequently investigated for formate production but often plagued with limited Faradaic efficiency (see Supplementary Fig. 6)^18,19,30^. In addition, we found that similar Bi nanosheets could be prepared from the cathodic reduction of BiOCl and BiOBr nanostructures (Supplementary Fig. 7). Electrochemical measurements revealed no noticeable difference in their formate selectivity or partial current density.

Finally, the operation durability of BiNS was assessed by bulk electrolysis at −1.5 V (*η* = 680 mV) for 10 h. Our catalyst delivered a stable cathodic current density of 15–16 mA cm^−2^ with no apparent sign of activity loss (Fig. 3d). Analysis of the formate in the catholyte after the 10 h electrolysis led to an incredible average Faradaic efficiency of ~95%, suggesting that the CO_2_RR selectivity was preserved during the long-term electrolysis. The 10 h durability test here was far from reaching the possible end of the catalyst life. Postmortem analysis after the long-term electrolysis revealed that BiNS preserved its composition and 2D morphology (Supplementary Fig. 8).

**Fig. 4:**
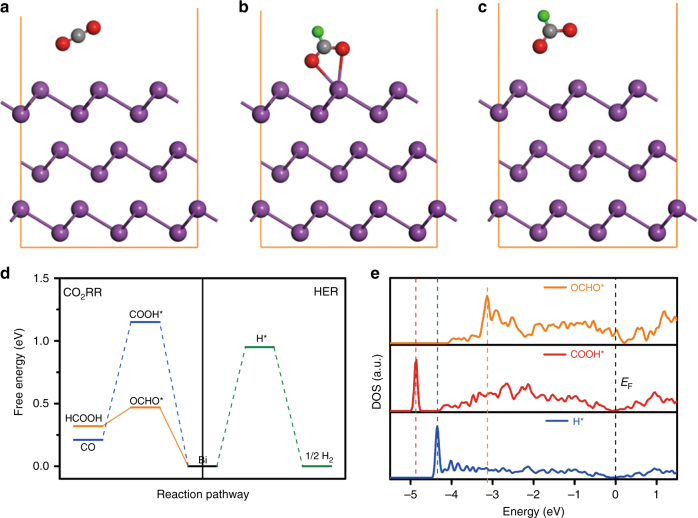
DFT simulation of the CO_2_RR process on Bi (001) plane. **a**–**c** Optimized geometric structure of **a** CO_2_, **b** OCHO* adsorbate and **c** HCOO^−^, where Bi, C, O, and H atoms were presented by purple, gray, red, and green spheres, respectively; **d** free-energy diagrams for HCOO^−^, CO, and H_2_ formation on Bi (001) plane; **e** projected *p*-orbital DOS of the Bi site with OCHO*, COOH*, or H* adsorbate, the Fermi level (*E*_F_) was at 0 eV, *E*_p_ in OCHO*, and COOH* and H* were highlighted with yellow, blue, and green dashed lines, respectively

To gain further insights into the excellent CO_2_RR performance of BiNS, DFT calculations were carried out via the computational hydrogen electrode methodology^31^. Rhombohedral Bi was represented using a trilayer structure in a 3 × 3 supercell. Its optimized lattice constants were *a* = *b* = 4.60 Å, consistent with experimental values. The simulation of CO_2_ reduction to formate was performed on the Bi (001) plane since it was the predominantly exposed crystal plane on BiNS. Optimized geometric structures of various states along the catalytic pathway were depicted in Fig. 4a–c, and corresponding energy profiles were summarized in Fig. 4d. CO_2_ reduction initiated with a proton-coupled electron transfer, leading to the protonation of C or O atom. Here, we found that the protonation of C atom to the OCHO* intermediate was mildly endothermic (+0.49 eV). Upon the second proton-coupled electron transfer that was exothermic (−0.17 eV), this intermediate transformed to HCOO^−^, and finally was spontaneously released from the catalyst surface as formate. By stark contrast, we found that the protonation of O atom in CO_2_ to COOH*—which was the intermediate to CO—was significantly uphill in energy (+1.16 eV), and that the free energy of H adsorption on Bi (001) was likewise too positive (+0.95 eV) to allow active HER. The binding strength of different intermediates could also be inferred from comparing *p*-projected density of states (DOS) of the active Bi site with adsorbates (Fig. 4e). The highest peak of active Bi DOS (*E*_p_) of OCHO* was the closest to the Fermi level, corresponding to the lowest filling of anti-bonding states and hence stronger adsorbate binding relative to COOH* and H*^32^. As a result, CO_2_ reduction to formate was the most energetically favorable among the three competing cathodic processes. It rationalized the observed high Faradaic efficiency for formate. The reaction overpotential was estimated to be 0.30 V and agreed reasonably well with our experimental finding (~0.4 V). Worth noting was that the binding strength of these three intermediates on Sn was reported to be relatively comparable, which thereby explained its inferior formate selectivity^33,34^.

### Coupling CO_2_RR and OER in full cells

**Fig. 5:**
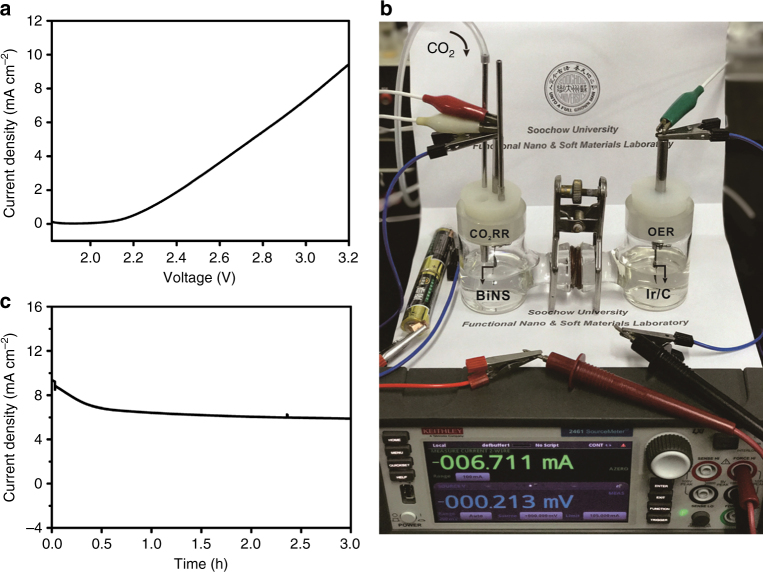
Full-cell electrolysis by coupling BiNS CO_2_RR with Ir/C OER. **a** Polarization curve for the full cell CO_2_RR-OER electrolysis; **b** photograph of the setup for the CO_2_RR-OER electrolysis powered by two AA-size alkaline batteries; **c** current evolution for the battery-powered CO_2_RR-OER electrolysis

Encouraged by the excellent CO_2_RR performance of BiNS in aqueous solution, we further pursued full-cell electrolysis to assess its practical implications. A total of 20 wt% Ir nanoparticles on Vulcan carbon black (Ir/C) was employed as the electrocatalyst for oxygen evolution reaction (OER). Despite its high cost, Ir/C was valued for its excellent OER activity at all pH conditions (particularly in neutral and acidic solution), and therefore chosen as the benchmark material to couple with BiNS CO_2_RR. Its OER polarization curve was first collected using the standard three-electrode setup and measured to reach 1 mA cm^−2^ at 0.82 V and 10 mA cm^−2^ at 0.99 V versus SCE (corresponding to an overpotential of *η*_1 mA cm−2_ = 0.34 V and *η*_10 mA cm−2_ = 0.51 V, respectively) in 0.5 M NaHCO_3_ (Supplementary Fig. 9). Full cells were then constructed by pairing BiNS cathode and Ir/C anode in a two-compartment cell, and their typical non-iR-compensated polarization curve was depicted in Fig. 5a. The CO_2_RR-OER reaction couple became turned on under the external voltage of ~2.1 V (in good agreement with the combined performance of these two electrocatalysts), and was able to deliver a current density of ~10 mA cm^−2^ at 3.2 V. Next, we used two AA-size alkaline batteries as the external power source (with open circuit voltage of ~3.1 V) to drive the CO_2_RR-OER electrolysis (Fig. 5b). A source-meter was connected in series to continuously monitor the current evolution. As shown in Fig. 5c, the current density started at ~8 mA cm^−2^, and after an initial decay over the first half an hour, roughly stabilized at 6 ~ 7 mA cm^−2^. We noted that the noticeable activity loss was mostly contributed from the anode side. The average Faradaic efficiency for formate during a typical 3 h full-cell electrolysis was measured to be ~95%. The electricity-to-formate conversion efficiency was calculated to be 47% based on the relative ratio of the stored chemical energy in formate to the electricity energy input (see Methods for details). Both efficiency values were remarkable. Based on these results, we estimated that if proper solar cells with matching *I–V* parameters and solar-to-electricity energy efficiency of 10–20% could be identified for powering the CO_2_RR-OER electrolysis in future, one could readily achieve an overall solar-to-fuel conversion efficiency of 5–10%. Here, the precious Ir/C OER electrocatalyst might also be replaced with non-precious metal based materials (such as CoPi)—of course at the price of significantly lower activity.

## Discussion

In summary, we reported that ultrathin BiNS could be prepared via the in situ topotactic transformation of BiOI nanosheet template under cathodic electrochemical environments. Resultant nanosheets preserved the extended 2D structure and single crystallinity. They processed enlarged surface area and abundant under-coordinated Bi sites. The topotactic transformation was understood based on the intrinsic structural correlation between BiOI and Bi, and supported by our theoretical simulations. When evaluated as the CO_2_RR electrocatalyst in 0.5 M NaHCO_3_, BiNS enabled high-performance CO_2_ reduction to formate with excellent selectivity (Faradaic efficiency >90% over a broad potential), activity (large HCOO^−^ partial current density of 24 mA cm^−2^ at −1.74 V) and durability (no loss in activity or selectivity for at least 10 h). Such a performance was more attractive than many other CO_2_RR electrocatalysts for formate production^19,20,30,35,36^. DFT calculations suggested that the high-reaction selectivity toward formate was due to its stabilized intermediate (OCHO*) on Bi (001) surface relative to COOH* or H*. At last, we demonstrated BiNS could be coupled with Ir/C to achieve efficient CO_2_RR-OER electrolysis when powered by two AA alkaline batteries. A Faradaic efficiency of ~95% and electricity-to-formate conversion efficiency of 47% were reported.

## Methods

### Preparation of BiOI nanosheets

In a typical synthesis, 0.97 g of Bi(NO_3_)_3_∙5H_2_O was first dissolved in 20 mL of 1.2 M glacial acetic acid and vigorously stirred for 20 min. It was then added dropwise with 1.66 g KI dissolved in 5 mL of deionized water, followed by the addition of 1 M NaOH for adjusting the solution pH to 6. Thus formed orange suspension was stirred for another 30 min before it was transferred to a 50 mL Teflon-lined autoclave and hydrothermally treated at 160 °C for 2 h. The final product was collected by centrifugation, repetitively washed with deionized water, and finally lyophilized.

### Material characterizations

XRD was performed on a PANalytical X-ray diffractometer. SEM images were taken using a Zeiss scanning electron microscope. TEM was conducted on a FEI Tecnai F20 transmission electron microscope operating at an acceleration voltage of 200 kV. AFM images were taken using a Veeco (MultiMode V) atomic force microscope.

### Electrochemical measurements

For the reductive transformation to BiNS, and subsequent CO_2_RR measurements, 1 mg of BiOI prepared above and 0.5 mg of Ketjenblack carbon were dispersed in 250 μL of ethanol and 6 μL of 5 wt% Nafion solution, and bath-sonicated for 30 min to form a uniform catalyst ink. The ink was then dropcast onto a 1 × 1 cm^2^ Teflon-treated carbon fiber paper (AvCarb P75 from Fuel Cell Store) and naturally dried. Commercial Bi nanopowder was purchased from Shanghai Macklin Biochemical Co. with a nominal purity of 99.99% and average particle size of ~200 mesh (corresponding SEM and XRD data were available in Supplementary Fig. 10). Its working electrode was prepared under the identical condition except for using 1 mg of Bi nanopowder for the catalyst ink preparation. Electrochemical experiments were carried out in a custom-designed gas-tight H-type electrochemical cell with a Nafion-117 proton exchange membrane as the separator. Catalyst loaded carbon fiber paper and saturated calomel electrode (SCE) were used as the working and reference electrode respectively and placed in the cathodic compartment; a Pt gauze counter electrode was used as the counter electrode and placed in the anodic compartment. Each compartment contained ~30 mL of 0.5 M NaHCO_3_ electrolyte, and their headspace was ~25 mL. The electrolyte was pre-saturated with N_2_ (pH = 8.4) or CO_2_ (pH = 7.4). CV and polarization curves were collected at a scan rate of 10 mV s^−1^ using CHI 660E potentiostat. All the potential readings were iR-corrected, and reported against SCE unless otherwise specified. For the complete topotactic transformation of BiOI to BiNS, the working electrode was biased at −1.55 V for 2 h. Resultant working electrode was immediately taken out of the electrolyte, briefly rinsed with deionized water and subjected to microscopic characterizations as described in the main text. For CO_2_RR electrocatalysis on BiNS or commercial Bi nanopowder, a flow of 20 sccm of CO_2_ was continuously bubbled into the electrolyte to maintain its saturation. For the full-cell measurements, 20 wt% Ir/C was used as the OER electrocatalyst and similarly loaded onto 1 × 1 cm^2^ carbon fiber paper electrode to achieve an areal loading of 1 mg cm^−2^. CO_2_RR-OER electrolysis was performed in the same two-compartment cell controlled by the potentiostat in the two electrode configuration or powered by two AA-sized alkaline batteries. Current density during electrolysis was measured by 2461-digital source-meter (Keithley). All electrochemical results for this part were non-iR-compensated.

### Product analysis

In order to determine the reduction products and their Faradaic efficiency, electrolysis was conducted at selected potentials for 1–2 h. Gaseous products in the headspace of the cathodic compartment were periodically vented into the gas-sampling loop of a gas chromatograph (GC, Aligent 7890B). The GC was equipped with a molecular sieve 5 A and two porapak Q columns. Nitrogen was used as the carrier gas. Reduction products were first analyzed by a thermal conductivity detector (TCD) for the H_2_ concentration, and then analyzed by flame ionization detector (FID) with a methanizer for CO. The concentration of gaseous products was quantified by the integral area ratio of the reduction products to standards. Their Faradaic efficiency was calculated as follows:

1 $${\rm FE}(\% ) = \frac{{Q_{{\rm co}}}}{{Q_{{\rm tot}}}} \times 100{\mathrm{\% }} = \frac{{\left( {\frac{v}{{60\,{\rm s/min}}}} \right) \times \left( {\frac{y}{{24,000\,{\rm cm^3/mol}}}} \right) \times N \times F \times 100{\mathrm{\% }}}}{j},$$

where *v* = 20 sccm is the flow rate of CO_2_, *y* is the measured concentration of product in 1 mL sample loop based on the calibration of the GC with a standard gas, *N* = 2 is the number of electrons required to form a molecule of CO or H_2_, *F* is the Faraday constant (96,500 C mol^−1^), *j* is the recorded current.

The liquid products were collected at the conclusion of each electrocatalysis, and first identified to only contain formate by NMR (Aligent DD2–600), and then quantitatively analyzed using an ion chromatograph (Dionex ICS-600). The concentration of formate was determined from its IC peak area using the calibration curve from a series of standard HCOONa solutions. Its Faradaic efficiency was calculated as follows:

2 $${\rm FE_{HCOO^ - }}\left( {\mathrm{\% }} \right) = \frac{{Q_{{\rm HCOO^ -} }}}{{Q_{{\rm tot}}}} \times 100{\mathrm{\% }} = \frac{{n_{{\rm HCOO^ - }} \times N \times F \times 100{\mathrm{\% }}}}{{j \times t}},$$

where *n*_HCOO−_ is the measured amount of formate in the cathodic compartment and *t* is the reaction time. The HCOO^−^ partial current density at different potentials was calculated by multiplying the overall geometric current density and its corresponding Faradaic efficiency.

The electricity-to-formate conversion efficiency of full cells was calculated as follows:

3 $$\eta = \frac{{\Delta rG_m^\theta \times n_{{\rm HCOO^ - }}}}{{Q_{{\rm tot}} \times V}},$$

4 $$\Delta _rG_m^\theta \left( {298.15\,{\rm K}} \right) = \Delta _fG_m^\theta \left( {{\rm HCOO^ - }} \right) + \frac{1}{2}\Delta _fG_m^\theta \left( {{\rm O}_2} \right) - \Delta _fG_m^\theta \left( {{\rm CO_2}} \right) - \Delta _fG_m^\theta \left( {{\rm H_2O}} \right),$$

where $\Delta rG_m^\theta ( = 270.138\,{\rm kJ \cdot mol^{ - 1}})$ is the energy gain during CO_2_RR, and *V* (~3 V) is the working voltage.

### Computation details

First-principle DFT calculations were carried out using the plane-wave technique with exchange–correlation interactions modeled by GGA-PBE^37^ functional, as implemented in the VASP code^38,39^. The ion–electron interactions were described by the projector-augmented plane-wave approach^40,41^. A plane-wave cutoff energy of 420 eV with Fermi-level smearing of 0.05 eV for slabs and 0.01 eV for gas-phase species was used in all calculations. The *k*-space samplings were set as 3 × 3 × 1 for the geometry optimization of Bi (001) slab, and 13 × 13 × 1 for the computation of electronic structure. The convergence thresholds of energy and forces were set as 1 × 10^−5^ eV and 0.02 eV Å^−1^, respectively. The Bi (001) slab was constructed with six atomic layers and a vacuum space of 15 Å along the *z*-direction, of which the top two layers were allowed to relax. The FPMD simulation was performed using an optimized 6 × 6 supercell of tetragonal Bi and NVT ensemble, where the time step was 1.0 fs and the temperature (300 K) was controlled by Nosé–Hoover method^42^.

The free energy (*G*) for each species was expressed as:

5 $$G = E_{{\rm DFT}} + E_{{\rm ZPE}} - { TS},$$

where *E*_DFT_, *E*_ZPE_, *S*, and *T* were electronic energy, zero point energy, entropy, and system temperature (298.15 K), respectively. For absorbates, *E*_ZPE_ and *S* were determined by vibrational frequencies calculations, where all 3N degrees of freedom were treated as harmonic vibrational motions without considering contributions from the slab. For molecules, those were taken from the NIST database^43^. The relevant contributions to *G* were listed in Supplementary Table 1. Note that the dipole and solvent corrections were also included in surface calculations. The solvent effect on adsorbates was achieved using the Poissson–Boltzmann implicit solvation model with a dielectric constant of 80^44^. Moreover, a series of gas-phase thermochemical reaction enthalpies (Supplementary Table 2) were tested to correct the *E*_DFT_ of CO_2_, CO, and HCOO^−^ due to the inaccuracy of the PBE functional to describe those molecules^31^. From the Supplementary Table 2, the energy errors for CO_2_, CO, and HCOO^−^ were about +0.17, −0.24, and +0.17 eV, respectively. Accordingly, +0.17 eV correction was applied in the electronic energies of CO_2_ and HCOO^−^, whereas −0.24 eV correction was applied to CO.

### Data availability

The data that support the findings of this study are available from the corresponding author upon reasonable request.

## Electronic supplementary material

Supplementary Information(PDF 1422 kb)
