# Supplementary material for: Ultrathin bismuth nanosheets from in situ topotactic transformation for selective electrocatalytic CO2 reduction to formate
Source: Nat Commun. 2018 Apr 3;9:1320. doi: 10.1038/s41467-018-03712-z (PMC5882965; doi:10.1038/s41467-018-03712-z)
Supplement: Supplementary file 1 — Supplementary Information(PDF 1422 kb) [file 41467_2018_3712_MOESM1_ESM.pdf]

Supplementary Information

Ultrathin Bismuth Nanosheets from In-Situ Topotactic Transformation for Selective Electrocatalytic Carbon Dioxide Reduction to Formate

By Han et al.

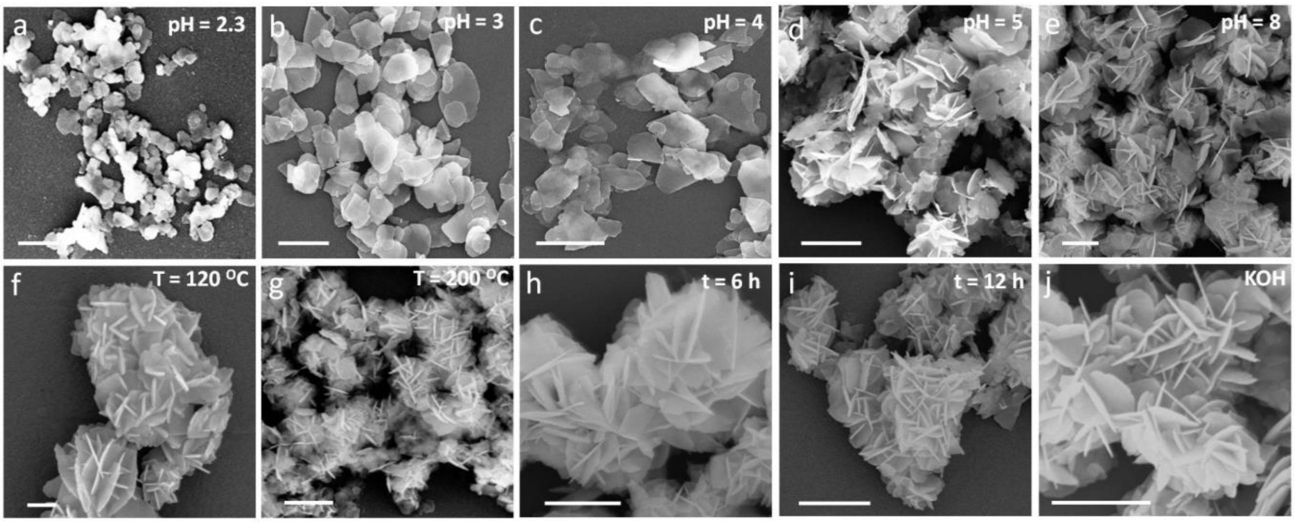

Supplementary Figure 1. SEM images of BOI products from different synthetic conditions. (**a-e**) different pHs, (**f,g**) different reaction temperatures, (**h,i**) different reaction times, and (**j**) KOH instead of NaOH, all under otherwise identical conditions as described in our Experimental Methods. Scale bar, 2 μm (**a-e**); 200 nm (**f**); 1 μm (**g-j**).

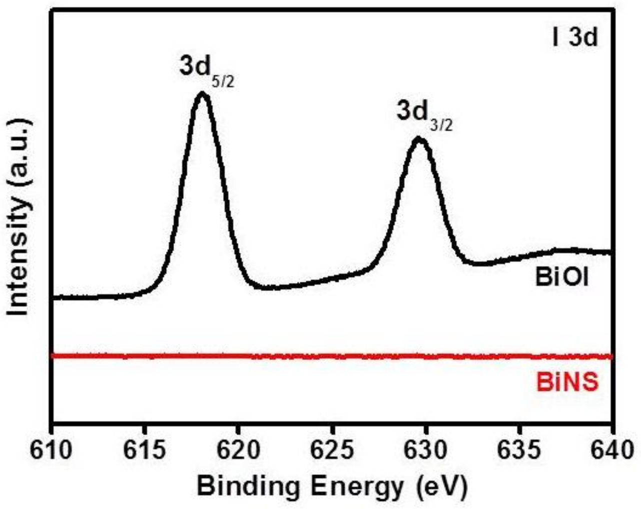

Supplementary Figure 2. I 3d XPS spectra of BiOI and BiNS.

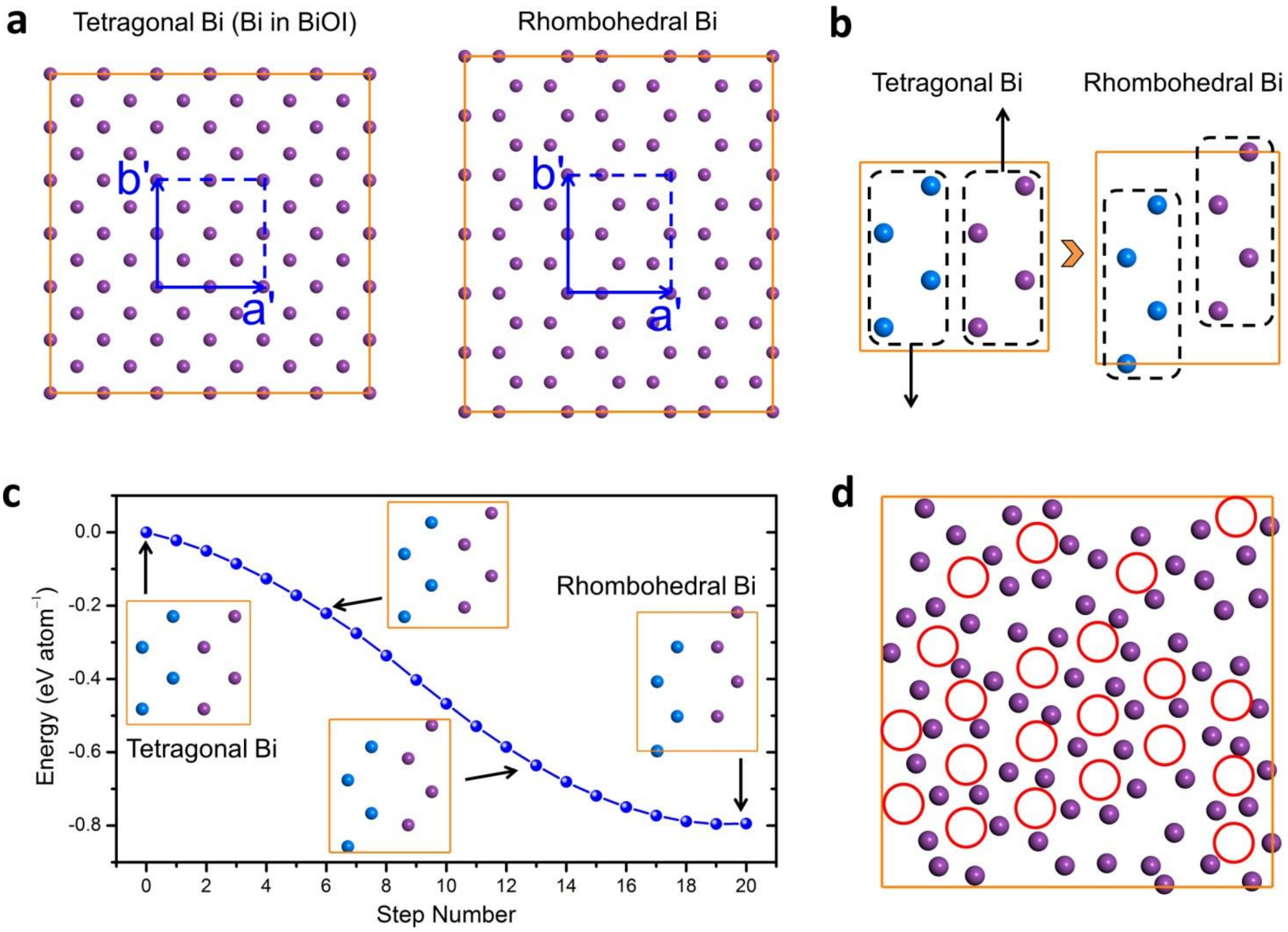

Supplementary Figure 3. Theoretical insights on the topotactic conversion from BiOI to Bi. (**a**) Arrangement of Bi atoms within the (001) plane of (left) tetragonal BiOI and (right) rhombohedral Bi; *a'* and *b'* represent the lattice vectors of supercells. (**b**) Schematic illustration showing the transformation from tetragonal Bi to rhombohedral Bi by sliding Bi atoms along the *b'*-direction. (**c**) Energy change during the transformation from tetragonal Bi to rhombohedral Bi, suggesting that once reduced, the conversion from BiOI to Bi is spontaneous. (**d**) Structural snapshot of original tetragonal Bi nanosheet after 1000 steps (1ps) of first-principles molecular dynamics simulation; red rings represent hexatomic Bi rings formed.

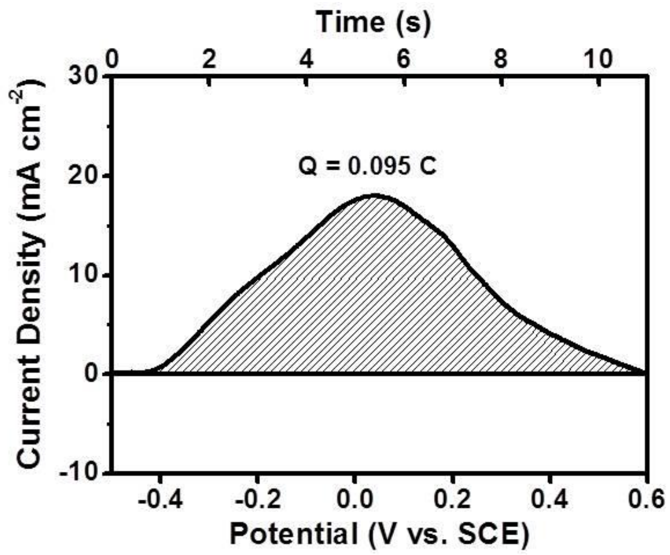

Supplementary Figure 4. Total charge integrated from the Bi^{3+}/Bi^{0} cathodic wave. Using this value, we further estimated the percentage of surface Bi sites.

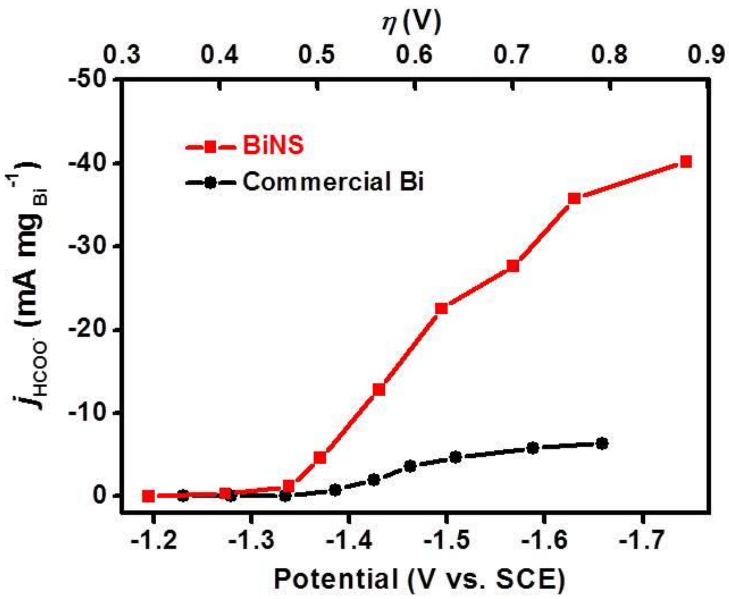

Supplementary Figure 5. Mass-specific formate partial current density of BiNS and commercial Bi nanopowder.

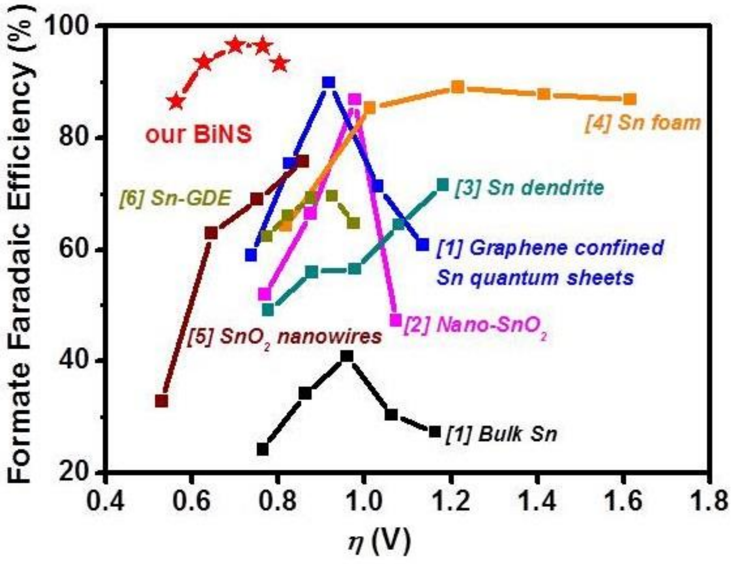

Supplementary Figure 6. Comparison on the formate Faradaic efficiency of BiNS and Sn-based CO_2RR electrocatalytsts. Refer to the Supplementary References at the end for more information.

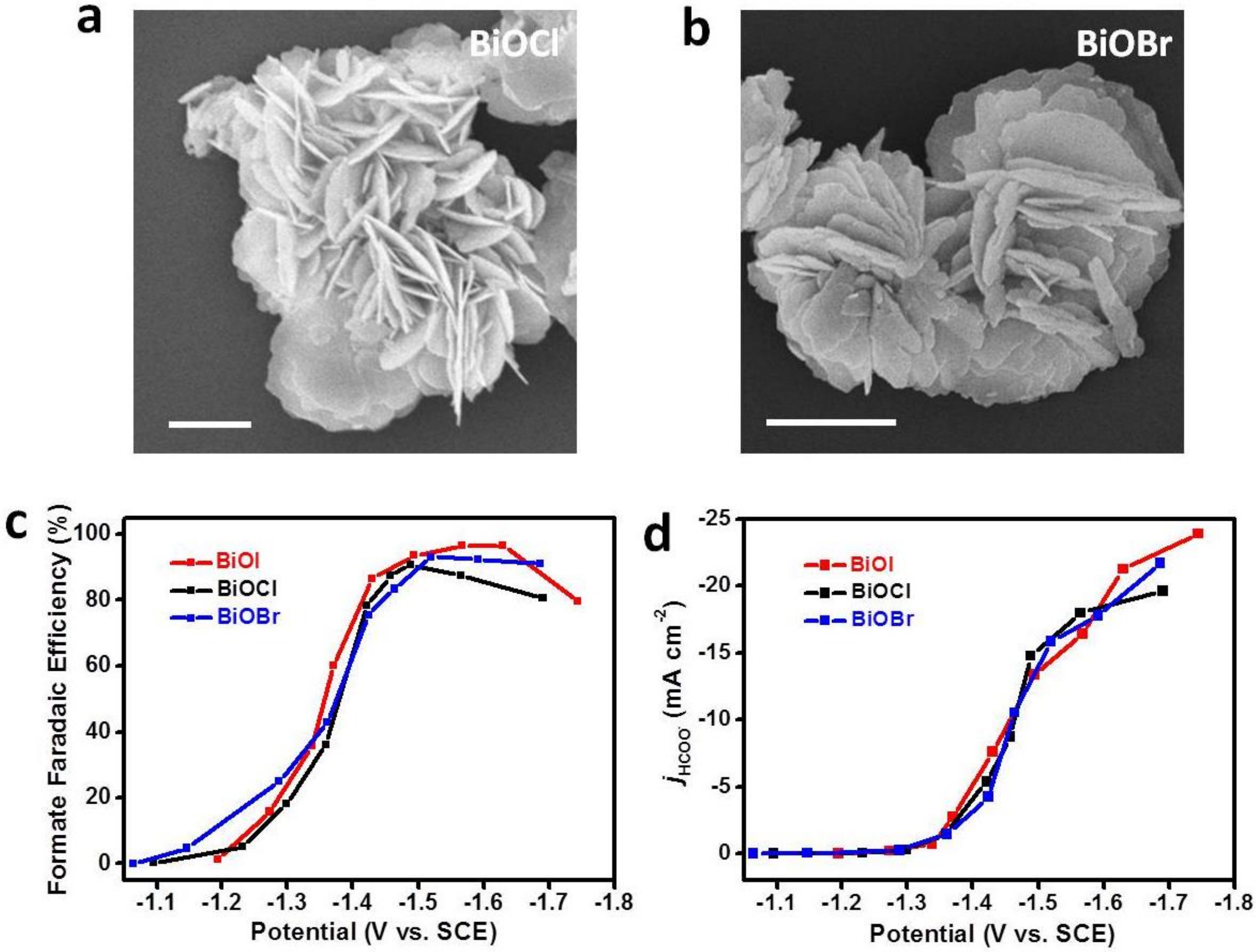

Supplementary Figure 7. The effect of different halides. (**a,b**) SEM images of BiOCl and BiOBr nanostructures using KCl or KBr as the halide precursor instead of KI under otherwise identical conditions as described in our Experimental Methods. (**c**) Formate Faradaic efficiency and (**d**) partial current density of Bi nanosheets derived from BiOCl and BiOBr in comparison with BiNS derived from BiOI. Scale bar, 1 μm (**a,b**).

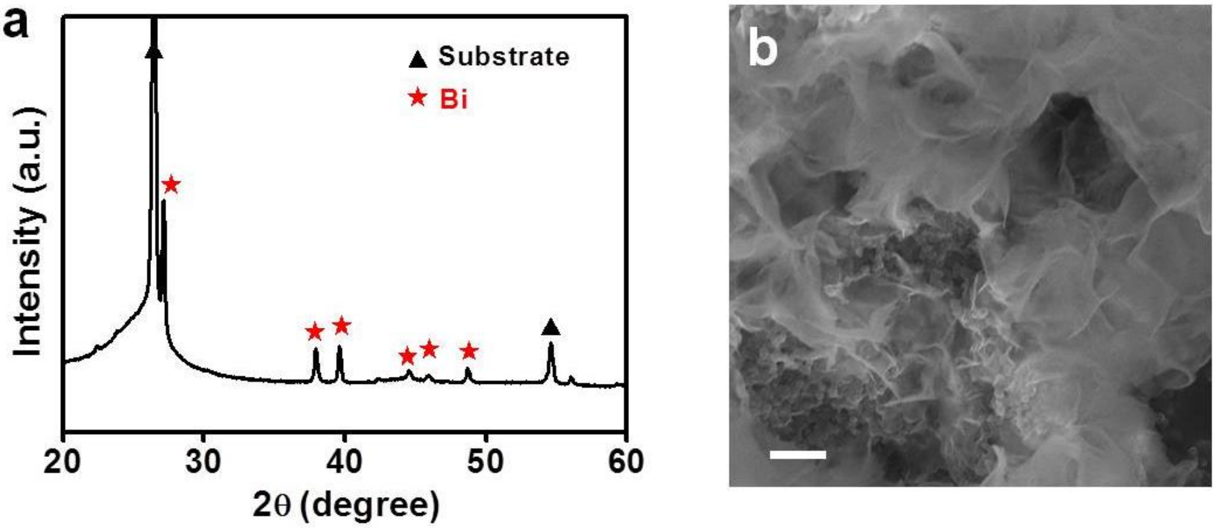

Supplementary Figure 8. Postmortem characterizations of BiNS after the amperometric stability test. (**a**) XRD pattern; (**b**) SEM image. Scale bar, 500 nm (**b**).

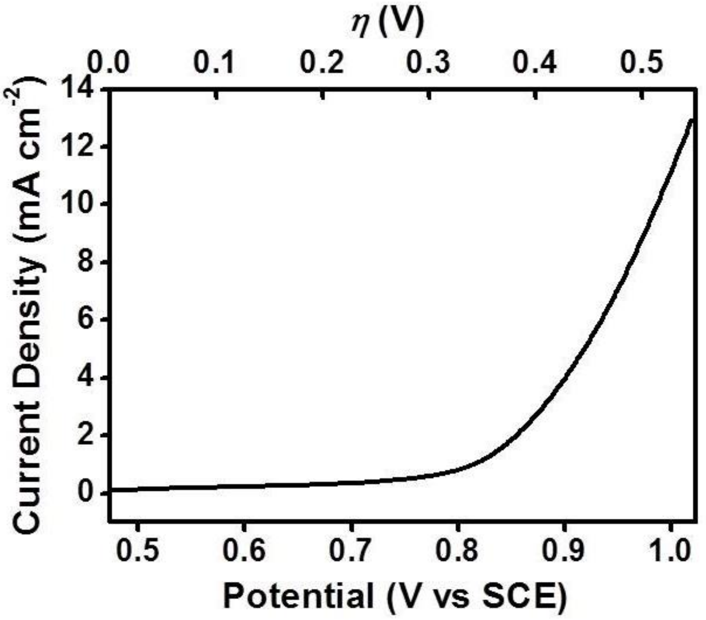

Supplementary Figure 9. Polarization curve of Ir/C for OER electrocatalysis in 0.5 M $NaHCO_3$.

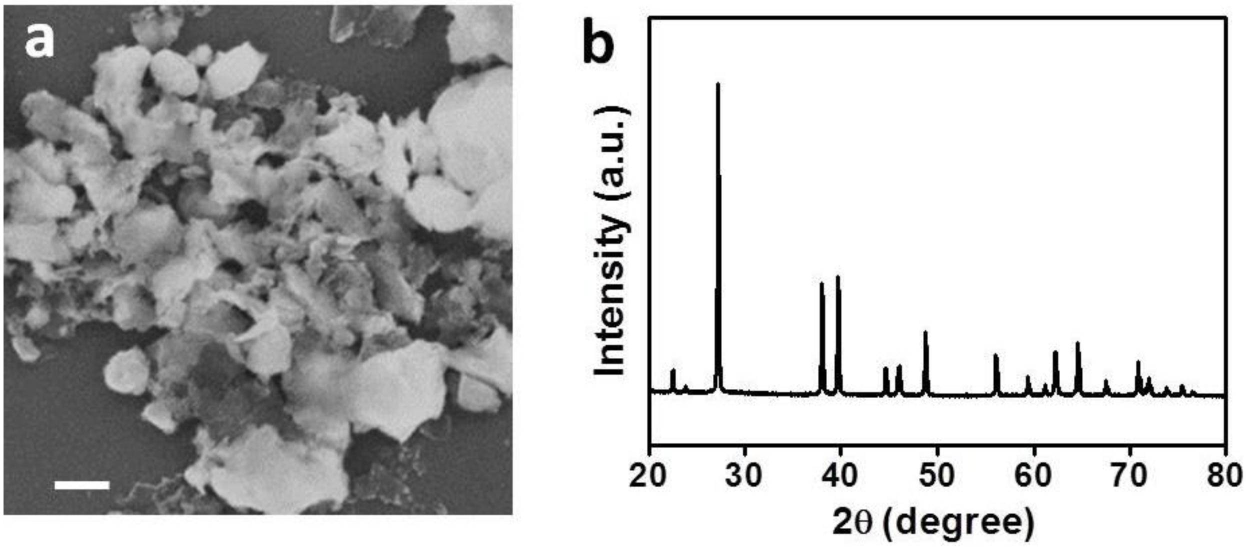

Supplementary Figure 10. Structural characterizations of commercial Bi nanopowder. (**a**) SEM image; (**b**) XRD pattern. Scale bar, 200 nm (**a**).

Supplementary Table 1. Zero-point energy correction (E_{ZPE}), entropy contribution (TS), and the total free energy correction ($G–E_{elec}$) for the studied system.

Species	E_{ZPE} (eV)	$-TS$ (eV)	$G–E_{elec}$ (eV)
**H_2**	0.27	–0.40	–0.13
**H_2O**	0.56	–0.67	–0.11
**CO_2**	0.31	–0.66	–0.35
**HCOOH**	0.89	–0.89	0.00
**CO**	0.13	–0.61	–0.48
**H***	0.14	–0.03	0.11
**COOH***	0.59	–0.29	0.30
**OCHO***	0.58	–0.33	0.25

Supplementary Table 2. Analysis of reaction enthalpies (in eV) of gas-phase thermochemical reactions. ΔH_{ref} was the literature values taken form NIST, ΔH_{cal} was PBE calculated reaction enthalpies, ΔH_{cor} was corrected reaction enthalpies that included −0.24 eV for CO and +0.17 eV for CO_2 and $HCOO^-$, and error was defined as the energy difference between ΔH_{cor} and ΔH_{ref}.

**Stoichiometry**	**ΔH_{ref}**	**ΔH_{cal}**	**ΔH_{ref}–ΔH_{cal}**	**ΔH_{cor}**	***Error***
**$CO_2+H_2 \rightarrow CO+H_2O$**	0.43	0.84	0.41	0.43	0.00
**$CO_2+4H_2 \rightarrow CH_4+2H_2O$**	−1.71	−1.48	0.17	−1.71	0.00
**$CO+3H_2 \rightarrow CH_4+H_2O$**	−2.14	−2.35	−0.24	−2.14	0.00
**$CO_2+H_2 \rightarrow HCOOH$**	0.15	0.15	0.00	0.15	0.00
**$CO+H_2O \rightarrow HCOOH$**	−0.27	−0.68	−0.41	−0.27	0.00
**$CO_2+3H_2 \rightarrow CH_3OH+H_2O$**	−0.55	−0.39	0.16	−0.56	0.01
**$CO+2H_2 \rightarrow CH_3OH$**	−0.98	−1.23	−0.25	−0.99	0.01
**$CO_2+3H_2 \rightarrow 1/2C_2H_4+2H_2O$**	−0.66	−0.43	0.17	−0.66	0.00
**$CO+2H_2 \rightarrow 1/2C_2H_4+H_2O$**	−1.09	−1.30	−0.24	−1.09	0.00
**$CO_2+7/2H_2 \rightarrow 1/2C_2H_6+2H_2O$**	−1.37	−1.14	0.17	−1.37	0.00
**$CO+5/2H_2 \rightarrow 1/2C_2H_6+H_2O$**	−1.80	−2.01	−0.24	−1.80	0.00

Supplementary References

1. Lei F., *et al.* Metallic tin quantum sheets confined in graphene toward high-efficiency carbon dioxide electroreduction. *Nat. Commun.* **7**, 12697 (2016).
2. Zhang S., Kang P., Meyer T. J. Nanostructured tin catalysts for selective electrochemical reduction of carbon dioxide to formate. *J. Am. Chem. Soc.* **136**, 1734-1737 (2014).
3. Won D. H., Choi C. H., Chung J., Chung M. W., Kim E. H., Woo S. I. Rational design of a hierarchical tin dendrite electrode for efficient electrochemical reduction of CO_2. *ChemSusChem* **8**, 3092-3098 (2015).
4. Du D., *et al.* Achieving both high selectivity and current density for CO_2 reduction to formate on nanoporous tin foam electrocatalysts. *ChemistrySelect* **1**, 1711-1715 (2016).
5. Kumar B., *et al.* Reduced SnO_2 porous nanowires with a high density of grain boundaries as catalysts for efficient electrochemical CO_2-into-HCOOH conversion. *Angew. Chem. Int. Ed.* **56**, 3645-3649 (2017).
6. Irtem E., *et al.* Low-energy formate production from CO_2 electroreduction using electrodeposited tin on GDE. *J. Mater. Chem. A.* **4**, 13582-13588 (2016).
